# Adaptive Computerized Working Memory Training in Patients With Mild Cognitive Impairment. A Randomized Double-Blind Active Controlled Trial

**DOI:** 10.3389/fpsyg.2019.00807

**Published:** 2019-04-12

**Authors:** Marianne M. Flak, Haakon R. Hol, Susanne S. Hernes, Linda Chang, Andreas Engvig, Knut Jørgen Bjuland, Are Pripp, Bengt-Ove Madsen, Anne-Brita Knapskog, Ingun Ulstein, Trine Lona, Jon Skranes, Gro C. C. Løhaugen

**Affiliations:** ^1^Department of Clinical and Molecular Medicine, Norwegian University of Science and Technology, Trondheim, Norway; ^2^Department of Pediatrics, Sørlandet Hospital HF, Arendal, Norway; ^3^Department of Radiology, Sørlandet Hospital HF, Arendal, Norway; ^4^Department of Clinical Science, University of Bergen, Bergen, Norway; ^5^Department of Medicine, The Memory Clinic Geriatric Unit, Sørlandet Hospital, Arendal, Norway; ^6^Department of Diagnostic Radiology and Nuclear Medicine, and Department of Neurology, University of Maryland School of Medicine, Baltimore, MD, United States; ^7^Department of Neurology, Johns Hopkins University School of Medicine, Baltimore, MD, United States; ^8^Department of Medicine, Diakonhjemmet Hospital, Oslo, Norway; ^9^Department of Research, Sørlandet Hospital, Arendal, Norway; ^10^Oslo Centre of Biostatistics and Epidemiology Research Support Services, Oslo University Hospital, Oslo, Norway; ^11^Department of Geriatric Medicine, The Memory Clinic, Oslo University Hospital, Oslo, Norway; ^12^Department of Psychiatry, Age Psychiatry, The Hospital of Telemark, Skien, Norway

**Keywords:** computerized cognitive training, working memory, mild cognitive impairment (MCI), neuropsychological outcomes, randomized controlled trial (RCT)

## Abstract

**Objective:**

We investigated if a 5-week computerized adaptive working memory training program (Cogmed^®^) of 20 to 25 sessions would be effective in improving the working memory capacity and other neuropsychological functions compared to a non-adaptive working memory training program (active-controlled) in adult patients with mild cognitive impairment (MCI).

**Methods:**

This randomized double-blinded active control trial included 68 individuals aged 43 to 88 years, 45 men and 23 women, who were diagnosed with MCI at four Memory clinics. The study sample was randomized by block randomization to either adaptive or non-adaptive computerized working memory training. All participants completed the training, and were assessed with a comprehensive neuropsychological test battery before the intervention, and at 1 and 4 months after training.

**Results:**

Compared to the non-adaptive training group, the adaptive training group did not show significantly greater improvement on the main outcome of working memory performance at 1 and 4 months after training.

**Conclusion:**

No difference were found between the two types of training on the primary outcome of working memory, or on secondary outcomes of cognitive function domains, in this sample of MCI patients. Hence, the hypothesis that the adaptive training program would lead to greater improvements compared to the non-adaptive training program was not supported. Within group analyses was not performed due to the stringent RCT design.

## Introduction

Mild cognitive impairment (MCI) is an intermediate stage between normal cognition and dementia. The risk for developing dementia for a person with MCI is 10 times higher than that for a cognitively normal person at the same age (Petersen et al., 1999; Petersen, 2009). Reduction of the working memory capacity is often associated with MCI (Saunders and Summers, 2010). Working memory refers to our ability to keep information “on line,” while we actively use the information (Eysenck, 1988; Baddeley and Hitch, 2000; Baddeley, 2003). A person’s working memory capacity is presumed to be a core function modifiable by repeated exposure to working memory tasks, such as the computerized adaptive working memory training. With adaptive training, the tasks become progressively more difficult as the participant improves, while the non-adaptive training uses the same fixed, lower demand level, for the tasks. The adaptive aspect is a feature of the training that can be considered a moderator of the hypothesized efficacy, which is based on principles of neuroplasticity (Constantinidis and Klingberg, 2016; Weicker et al., 2016). Also older adults exhibit neural and cognitive plasticity (Vaynman et al., 2004; Engvig et al., 2014; Bergmann et al., 2015). The plasticity paradigm is often held as the rationale or logic behind cognitive training (Li et al., 2004; Schaie, 2005).

Computerized cognitive training programs or “brain games,” including Cogmed^®^, has gained commercial popularity as possible non-pharmacological interventions aimed at cognitive enhancement. These training programs have been applied to healthy older adults, and recently also for clinical populations, such as those with MCI. However, the effectiveness of “brain training” programs had been controversial (Rabipour and Raz, 2012; Allaire et al., 2014; Makin, 2016). The Cogmed program was selected as intervention method as it provided a placebo version of the training adequate for our RCT-design. As with most cognitive training methods, Cogmed has been debated regarding effect that varies across studies and clinical groups. Positive effects, especially on transfer tasks, were shown in very preterm born children (Løhaugen et al., 2011; Grunewaldt et al., 2013) and human immunodeficiency virus-seropositive patients (Chang et al., 2017), while others found little or mixed effect of transfer in children with ADHD (Egeland et al., 2013), young and older adults, and individuals in recovery from stroke (Shipstead et al., 2012).

However, the tendency for not publishing negative results in clinical trials is also relevant for the field of computerized cognitive training, including the use of the Cogmed program. Hence, publication bias may contribute to distort the literature and knowledge base on results of computerized cognitive training (Joober et al., 2012).

In 2016, an authoritative review of the literature titled *Do “brain training” programs work*? was published, initiated by the Association for Psychological Science (APS) since a comprehensive review of the brain training literature that weighted on the quantity and quality of the evidence was lacking (Simons et al., 2016). The review provides best practice guidelines regarding the study designs and recommends double-blinded, placebo-controlled randomized trials to evaluate the efficacy of brain training. The review indicates that many of the published intervention studies had methodological problems with design and analysis, and stated several recommendations to mitigate these problems.

Several major studies have demonstrated that cognitively unimpaired older adults could benefit from cognitive enhancement through repeated practice on cognitive tasks. For example, the Improvement in Memory with Plasticity-based Adaptive Cognitive Training (IMPACT) study found that a computerized cognitive training program improved measures of memory and attention in older adults more than the control program (Smith et al., 2009). Further, investigators of the Advanced Cognitive Training for Independent and Vital Elderly (ACTIVE) group conducted a cognitive intervention study involving cognitively non-impaired senior individuals (Jobe et al., 2001). The study participants were randomly assigned to four different groups: a memory-training group, a reasoning or problem-solving group, a processing speed training group, and a no-contact control group. All three intervention groups showed improvements in cognitive function after training compared to their baseline function. The greatest gains were seen in processing speed, followed by improvement in reasoning domain and in memory functions. Furthermore, the gains were maintained years after the interventions (Jobe et al., 2001; Ball et al., 2002; Willis et al., 2006; Unverzagt et al., 2009).

One key question is whether older adults with impaired cognitive function can improve their cognitive function using cognitive training programs in general. A variety of computerized cognitive training are feasible and promising interventions for individuals with MCI. However, conflicting findings are reported in the literature regarding the impact of cognitive training both in individuals with MCI and in those with early Alzheimer’s disease (AD). A review by a Cochrane group showed no significant effects of cognitive training among individuals with early stage AD (Clare et al., 2003). Other studies (Belleville et al., 2006; Rozzini et al., 2007; Talassi et al., 2007) and meta-analysis have found that cognitive interventions in MCI may have beneficial impacts on cognitive outcomes (Belleville, 2008; Mowszowski et al., 2010; Li et al., 2011; Chandler et al., 2016; Hill et al., 2017). A second question is, if computerized cognitive training is effective, will adaptive training paradigms be more effective that non-adaptive paradigms? A recent randomized controlled trial of older adults with risks for dementia found that computerized cognitive training with adaptive difficulty appeared to be superior to a more generic approach on both cognitive and non-cognitive outcomes (Bahar-Fuchs et al., 2017). A study from the Netherlands that used computerized adaptive working memory training of older adults with MCI suggests that working memory may improve with training (Vermeij et al., 2016), and another study found that adaptive training outperformed the placebo training on Span board, one of the three core cognitive measures (Hyer et al., 2016). However, there is no randomized controlled double-blinded study that has investigated if adults with MCI diagnosed in a memory clinic will benefit from an adaptive cognitive training program specifically targeting the working memory capacity. Overall, the optimal type of cognitive intervention for cognitively impaired individuals remains unclear. More specifically, whether the adaptive aspect is necessary to achieve beneficial effects from cognitive training in patients with MCI is unknown. The focus of the current study is to use a non-pharmacological intervention that can be classified as a “working memory building” technique: computerized adaptive training targeting working memory. The training is developed with the intention to normalize or improve the impaired function through repetitive tasks over a structured period of time. Further, we conducted a study that included the methodological rigor recommended by the APS panel.

The primary aim of our study was to investigate if training effects differed in groups of patients with MCI who trained with an adaptive versus a non-adaptive (active control) version of a computerized working memory training program (Cogmed^®^). Both training programs included 20–25 training sessions over 5 weeks. Primary outcome measures were non-trained working memory tasks and secondary outcome measures were other cognitive tasks measured by a battery of standardized neuropsychological tests. We hypothesized that the adaptive training program would lead to greater improvements on these outcome measures compared to the non-adaptive training program. The outcomes were assessed at two time points: 1 and 4 months after Cogmed^®^ training.

## Methods

We performed a double blind, placebo (active)-controlled, randomized clinical trial approved by the Norwegian Regional Committee for medical and health research ethics, South-Eastern region (2013/410/REK Sør-øst). Informed consents were obtained from each participant. The study has been conducted in accordance with the CONSORT 2010 statement for non-pharmacological interventions as thoroughly as possible. The study was pre-registered at ClinicalTrials.gov (NCT01991405) in November 2013 and updated in February 2019. Sample size and power calculation were done by using pre-training and post-training test scores obtained by Brehmer et al. (2012), where 72 patients were needed for inclusion in the study to obtain a statistical power of 80%. Allowing for a dropout rate of 20%, the number of participants needed for inclusion was set to 90 patients with MCI. The power calculations were based on the primary outcome measures of three neuropsychological tests on working memory. The statistical power measures were calculated using Sample Power 2.0. However, the number of participants included in the present trial is lower than the preregistered *n* = 90. The inclusion of participants was more time consuming than planned, and due to the time frame of the study data inclusion phase was closed after 4 years of duration.

As encouraged in the CONSORT 2010 statement, we published the protocol for the trial at the initial phase of the study (Flak et al., 2014).

### Participants

Participants were recruited from four memory clinics in Norway [Sørlandet Hospital, Arendal, The Hospital of Telemark, Oslo University Hospital and Diakonhjemmet Hospital, Oslo]. In order to minimize incidence/prevalence bias, the participants were recruited from those who were diagnosed with MCI during the last 15 months before study start (Sackett and Gent, 1979) and forward.

When patients were referred to a hospital Memory clinic due to memory complaints or other cognitive difficulties, patients are diagnosed by multidisciplinary teams. The assessment includes neuropsychological tests, questionnaires for risk factors ascertainment, psychiatric conditions including depression, and MRI of the brain as specified by the Norwegian national guidelines (Norcog), and diagnosed. These guidelines are based on the Petersen/Winblad criteria. The MCI criteria were: (1) memory complaint, preferably confirmed by an informant, (2) impaired memory adjusted for age and education, (3) preserved general cognitive function, (4) overall intact activities of daily living, and (5) absence of dementia, in accordance with the ICD-10/DMS-IV criteria (Petersen, 2004). None of the participants were considered severely or moderately depressed according to their pretrial screenings. Socioeconomic status was calculated using Hollingshead’s index of education and occupational position, scaled from 1 (low) to 5 (high) (Hollingshead and Redlich, 2007).

The individuals that did not have a pc and/or an internet connection available, were provided with these resources for the duration of the training.

Table 1 describes the baseline characteristics and general cognitive ability scores of the participants.

**Table 1:** Baseline characteristics and cognitive scores in the two randomized groups, Mean (SD or range).

	Adaptive training *n* = 35	Non-adaptive training *n* = 34
Age at baseline assessment, years	65 (51–83)	67 (43–88)
Men/women	24/11	22/12
Education, years	13.6 (7–18)	12.8 (8–20)
Socioeconomic status	3.3 (1.2)	3.5 (1.2)
Full Scale Intelligence Quotient^∗^	97 (13)	96 (14)
General Ability Index^∗^	101 (14)	101 (16)
Verbal comprehension Index^∗^	101 (14)	99 (15)
Perceptual organization Index^∗^	101 (16)	103 (15)
Working memory Index^∗^	92 (13)	91 (14)
Processing speed Index^∗^	94 (14)	94 (18)

*MCI, mild cognitive impairment; SD, standard deviation. ^∗^Scales and indices from WAIS-IV, Wechsler Adult Intelligence Scale, 4^th^ edition.*

### Randomization

Permuted block randomization stratified by center (four centers) was used^1^. A statistician unrelated to the study provided the training group allocation for each participant (adaptive or non-adaptive training). The participants and the neuropsychologist performing all the assessments were unaware of the group allocation.

### Neuropsychological Assessment

The participants were assessed at three time points (T0: baseline, T1: 1 month after intervention, and T2: 4 months after intervention). The baseline assessment was completed before randomization. The cognitive evaluation included the administration of standardized and commonly used neuropsychological tests (Wechsler Memory Scale 3.ed/WMS-III, Delis–Kaplan Executive Function System/D-KEFS, California Verbal Learning Test 2.ed/ CVLT-II, and Rey Complex Figure Test/RCFT). We grouped the tests into the cognitive domains of working memory, attention, processing speed, visual and verbal learning, visual and verbal memory and executive functions (Rog and Fink, 2013). The outcomes are operationalized theoretical construct of the various cognitive domains. We used two or more tests, or subtests, as outcomes in each construct/domain. In addition, intelligence (Wechsler Adult Intelligence Scale 4.ed/WAIS-IV) was measured at baseline. We used alternative versions of the tests when available (i.e., Digit span, California Verbal Learning test- II and Verbal Fluency). Two of the tests that are included in our primary outcome/working memory domain: Spatial span and Letter number sequencing are both included in the MATRICS test battery (Measurement and Treatment Research to Improve Cognition in Schizophrenia) that are considered suitable for repeated testing in treatment effects research, since they are not expected to have learning, or test–retest effects^2^.

### Primary Outcome Measures (Working Memory Tasks)

Primary outcome measures were the scores on the working memory tasks WAIS-IV Digit Span backward, WMS-III Spatial Span backward, WMS-III Letter-Number Sequencing (see the working memory domain in Table 2).

**Table 2:** Cognitive constructs/domains and corresponding neuropsychological tests for the primary and secondary outcome measures.

Cognitive domain	Neuropsychological tests
**Primary outcome measures:**	
*Working memory*	WMS-III Digit Span backward, WMS-III Spatial Span backward, WMS-III Letter-Number Sequencing, CVLT-II Trial B
**Secondary outcome measures:**	
*Attention domain*	Digit Span forward, Spatial span forward, CVLT-II Trial 1,
*Processing speed*	D-KEFS Color Word Interference Test 1 color naming, D-KEFS Color Word Interference Test 2 Word reading
*Visual learning/short delay recall*	RCFT Immediate recall, WMS-III Faces I
*Visual memory/long delay recall*	RCFT Delayed Recall, WMS-III Faces II Delayed recall
*Verbal learning/short delay recall*	WMS-III Logical Memory I, CVLT-II Total learning, CVLT-II Short Delay Free Recall
*Verbal memory/ long delay recall*	Logical memory II Delayed recall, CVLT-II Long delay free recall
*Verbal memory, recognition*	CVLT Total hits
*Executive function*	RCFT, D-KEFS Color Word Interference Test 3 Inhibition, D-KEFS Color Word Interference test 4 Inhibit/Switching, D-KEFS Verbal Fluency Test Letter fluency, D-KEFS Verbal Fluency Test Category fluency, D-KEFS Verbal Fluency Test Category switching

*WMS-III, Wechsler Memory Scale 3.ed; D-KEFS, Delis–Kaplan Executive Function System; RCFT, Reys Complex Figure Test; CVLT-II, California Verbal Learning Test 2.ed.*

### Secondary Outcome Measures (Other Cognitive Function Tasks)

Secondary outcome measures were test performance in the following neuropsychological domains: attention, processing speed, visual episodic learning/short delay recall, visual episodic memory, long delay recall, verbal episodic learning/short delay recall, verbal episodic memory/long delay recall, verbal episodic memory, recognition domain, and executive function domain (Table 2).

We made some changes to the endpoints in the initial study protocol, but did not change any end point based on the data. The protocol included a pre-specification of primary and secondary outcomes. The primary outcome was originally the Spatial span board test assessing visual working memory, but were expanded to include Letter Number Sequencing and Digit Span Backwards, two auditory tests of working memory. These three test scores were grouped into a composite score, or working memory domain score, as primary endpoint. Secondary outcomes were originally neuropsychological test performance on a battery of neuropsychological tests, magnetic resonance imaging scores, and scores on questionnaires (self-report and informant report) of executive function in daily life, BRIEF-A and BADS. The neuropsychological battery and the Vineland questionnaire were quite time consuming for our participants with MCI. Therefore, we reduced the number of tests and deleted the Vineland questionnaire.

### Intervention: Adaptive or Non-adaptive Training

The intervention method used was a computerized and standardized adaptive working memory training program (Cogmed^®^^3^), developed by researchers at the Karolinska Institute, Stockholm, Sweden (Klingberg et al., 2005; Klingberg, 2010). This training program comprises several different “games” that require visuospatial working memory (remembering the position of objects) and a combination of verbal and visual working memory (remembering phonemes, letters, and digits). The physical appearance of the cognitive games is identical in the two versions of the program – the adaptive and the non-adaptive version. The only difference is the level of difficulty of the cognitive tasks. In the adaptive version, the tasks becomes more complex and difficult as the individual masters each level, making the participant work at his or her maximum capacity at all times, i.e., adaptive training. In the non-adaptive version, the participants trained at a fixed low level of difficulty, in which the span of each task did not exceed three items. The different tasks in the program are described in detail in the Supplementary Material.

During training, all participants received phone calls, at least once a week, to follow up on their progress and to motivate them; the calls were made by one of the researchers who followed the participants’ training through an online secured site. Both intervention groups followed the standard protocol (30–40 min of training per day, 5 days per week for 5 weeks). However, seven individuals needed a higher degree of coach support due to their inexperience with computers. We considered the training completed if the participants finished 20 or more of 25 training sessions. Both groups received the same rewards in the form of verbal praise from the program for completion of the training.

### Statistical Analysis

Data were analyzed with SPSS version 25 (IBM, Armonk, NY, United States) and Stata/SE 15.0 for Windows (StataCorp LLC, College Station, TX, United States).

In order to compare the intervention effects between groups, we used a linear mixed model for repeated measurements with a random intercept for each subject and maximum likelihood estimation. The model included the randomized group, the follow-up time, the baseline score of the outcome and the interaction terms between the randomized group and the follow-up time and between the baseline score of the outcome and the follow-up time as fixed effects. The mean difference between the randomized groups at 1 and 4 months follow-up was obtained using linear combinations of estimators after the model estimation.

### Neuropsychological Domains Based on Z-Score

We calculated z-scores in order to compare cognitive performance across subtests and domains. The z-scores were based on the mean score of the neuropsychological test results at baseline assessment divided by the standard deviation of the two groups (adaptive group and the placebo group) combined [z = (x-mean baseline)/SD]. In order to reduce the number of outcome variables, the z-scores of the subtests were clustered into nine theory-derived neuropsychological/cognitive domains: working memory domain as the primary outcome, and the other domains as secondary outcomes. An alpha level < 0.005 was considered statistically significant after Bonferroni-adjustment for multiple comparisons.

See also the Supplementary Material for the raw scores of each of the neuropsychological subtests within the domains. For these data, an alpha level < 0.001 was considered statistically significant after Bonferroni-adjustment.

## Results

**FIGURE 1:**
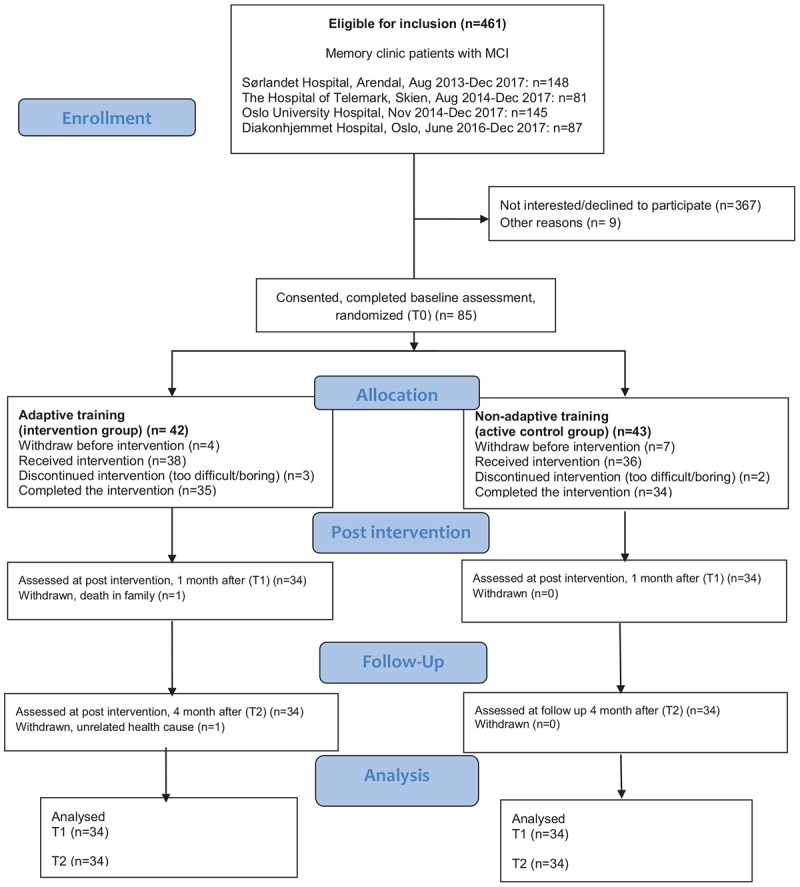
Flow chart of the recruited patients and the numbers of participants that completed the training (Consolidated Standards of Reporting Trials/CONSORT).

Eligible patients with MCI were invited to participate between August 2013 and December 2017 at Sørlandet Hospital, Arendal, between August 2014 and December 2017 in the Hospital in Telemark, between November 2014 and December 2017 at the Oslo University Hospital, and June 2016 – December 2017 at Diakonhjemmet Hospital, Oslo. A total of 461 patients were diagnosed with MCI (ICD-10 F06 and F07) in the inclusion periods and 85 of these patients were recruited as study participants. These 85 individuals went through baseline assessment of neuropsychological tests and MRI, and were then randomized to the adaptive intervention group or the non-adaptive placebo group. Eleven participants withdrew before they started training, and five participants withdrew after trying the program and finding it too difficult or boring. Sixty-eight individuals aged 43 to 88 years, 45 men and 23 women, completed the training program and all three clinical assessments. One patient was lost to the post-intervention assessment, and one patient was lost to the follow up assessment (Figure 1).

The classification of MCI subtypes was performed after inclusion. The study neuropsychologist categorized the MCI participants into amnestic and non-amnestic MCI based on their scores on the neuropsychological tests at baseline. Subjects with scores more than −1.5 SD from mean compared to norms on the tasks within the verbal and/or visual episodic memory domain were classified as having amnestic MCI. Normal scores in memory domains combined with scores more than −1.5 standard deviation from the mean in one or more of the other domains, resulted in categorization as non-amnesticMCI (Petersen et al., 1999, 2001; Winblad et al., 2004). Table 3 shown the distribution of the different MCI subtypes.

**Table 3:** Mild cognitive impairment subtype classification within the two training groups.

MCI subtype classification	Adaptive working memory training group, *n* = 34		Non-adaptive working memory training (placebo) group, *n* = 34	
	Frequency	Percent	Frequency	Percent
Amnestic single domain	5	14	4	11
Amnestic multiple domains	11	32	15	44
Non-amnestic single domain	14	41	13	38
Non-amnestic multiple domains	4	11	2	5
Total	34	100	34	100

Table 3 display the classification of MCI subtypes within the groups.

Means and standard deviations of the neuropsychological test results on the subtests for the adaptive training group the placebo training group are displayed in Supplementary Table S1.

Adaptive versus non-adaptive training, domain scores, no significant difference in gain was observed between the adaptive training group and the active-control (non-adaptive) training group between baseline and 1 month (T1–T0) and between baseline and 4 months (T2–T0) after training, neither on the primary outcome measures (working memory domain tests) or the secondary outcome measures (other cognitive domains) (Table 4).

**Table 4:** Mean differences between groups.

Z-score domain scores:	One month after training			Four months after training		
	Group difference	*p*-Value	95% CI	Group difference	*p*-Value	95% CI
**Working memory domain (main outcome):**						
	0.36	0.81	(−0.27 to 0.34)	0.17	0.27	(−0.13 to 0.48)
**Attention domain:**						
	3.85	0.51	(−7.79 to 15.49)	−0.30	0.95	(−11.94 to 11.33)
**Processing speed domain:**						
	3.81	0.31	(−3.58 to 11.21)	3.73	0.32	(−3.66 to 11.14)
**Visual learning, short delay recall domain:**						
	0.12	0.95	(−4.12 to 4.38)	4.08	0.06	(−0.16 to 8.34)
**Visual memory, long delay recall:**						
	4.37	0.24	(−3.03 to 11.78)	3.93	0.29	(−3.49 to 11.37)
**Verbal learning, short delay recall domain:**						
	−0.00	1.00	(−14.74 to 14.73)	3.47	0.64	(−11.25 to 18.21)
**Verbal memory, long delay recall domain:**						
	7.43	0.42	(−10.62 to 25.50)	10.81	0.24	(−7.25 to 28.87)
**Verbal memory, recognition domain:**						
	3.85	0.61	(−11.25 to 18.95)	6.90	0.37	(−8.20 to 22.00)
**Executive function domain: adjusted alpha level:**						
	3.70	0.39	(−4.86 to 12.27)	8.34	0.05	(−0.22 to 16.91)

*p-Values are from Linear mixed model for repeated measurements.*

In addition, we performed between group analyses of raw scores from the individual tests that comprised the neuropsychological domain scores. One task from the verbal episodic learning domain (California Verbal Learning-II, Short delay free recall) and one task from the verbal episodic memory domain (California Verbal Learning-II, Long delay free recall) showed greater increase in the scores in the adaptive training group compared to those in the non-adaptive training group at 1 month after training (T1–T0, learning-*p* = 0.003 and memory-*p* = 0.005). The scores on the California Verbal Learning Test-II, Short delay free recall also showed greater improvement in the adaptive training group compared to the non-adaptive group between baseline and 4 months after training (*p* = 0.006). However, after correction for multiple comparison, none of these results remained significant. We included these results in Supplementary Table S2.

## Discussion

In this group of MCI patients, no significant differences in the training effects were found between individuals that had the adaptive and those that had the non-adaptive working memory training on composite scores of working memory and other neuropsychological domains. Trends toward improved scores in the adaptive training group versus the non-adaptive training group on three tasks assessing verbal episodic learning and memory were not significant after correction for multiple comparisons. Hence, the hypothesized superior effect of the adaptive training program on working memory outcomes was not confirmed. The panel assembled by the APS classifies brain-training programs in multiple categories. The description of the one that includes the Cogmed^®^ program is “Brain-training companies citing multiple publications reporting tests of the effectiveness of a marketed brain-training product.’ The other categories are described as “Brain-training companies citing some intervention research, but not necessarily tests of the effectiveness of a marketed brain-training product,” “Brain-training companies citing no peer-reviewed evidence from intervention studies,” and “Companies conducting training or promoting products that fall outside the scope of this review” (Simons et al., 2016, p. 113; Table 1). Based on this categorization, we focused on comparing our findings with studies that have targeted MCI patients using training programs that cited multiple peer reviewed publications for effectiveness.

Since Cogmed^®^ focuses on training auditory and visual working memory, we had expected the tests in the working memory domain, with near transfer effects, to show greater training effects in the adaptive group than the non-adaptive group. However, the adaptively trained group did not show greater improvements than the non-adaptive group on the working memory tasks. The only tasks that showed trends toward greater adaptive training effect than non-adaptive training were the verbal episodic learning and memory tasks. The adaptively trained group tended to perform better on both learning (encoding) and delayed recall (retrieval) on a word list task. This meant that the patients who trained with the adaptive condition were able to learn and remember more words than those who trained with the non-adaptive program, even though the results did not reach significance after comparing for multiple comparisons. Encoding and retrieval are both skills that are known to deteriorate early in amnestic MCI patients, and poor results on these tasks are often an early marker of an underlying Alzheimer pathology (Petersen et al., 1999, 2018; Ivnik et al., 2000; Petersen, 2009; Bondi and Smith, 2014; Bondi et al., 2014). It would be interesting to investigate the data further and explore how the correlations between cognitive impairment severity, cognitive reserve and brain pathology burden affect individual training gains.

However, our findings did not support the hypothesis that adaptive training would outperform non-adaptive training. Our findings contrast with other studies that utilized adaptive training, which increases the load of the individual tasks as the participant improves. Bahar-Fuchs et al. (2017) reported improved cognitive outcomes on memory, learning, and global cognition at follow-up in MCI subjects. Belleville et al. (2018) found a significant effect on a delayed memory score, which lasted 6 months after training. These studies were not directly comparable to ours as Bahar-Fuchs and colleagues focused on training several cognitive functions and the study by Belleville and colleagues employed a therapist- and strategy-based memory training program while we focused on computerized training of the single domain of working memory. Yet, both these studies and ours included types of training with either increasing load or learning strategies for the participants individually and continuously adjusted. However, their studies both include programs that target multiple cognitive domains, which may be more effective than training one single domain in an MCI sample since the severity of the cognitive impairments may affect multiple domains in this heterogeneous condition.

The first study that evaluated individuals with MCI using the same training program (Cogmed) also included a non-adaptive training condition as we did in our study (Hyer et al., 2016). Contrary to our findings, they noted that the adaptive training significantly outperformed the non-adaptive version of the program on a primary outcome measure (Span board task). Hyer and colleagues reported that during the first week of training, the investigators became aware of the group allocation since subjects in the non-adaptive training group reported the ease of training. Such unblinding of the training type did not occur in our study, as several of the participants struggled even with the non-adaptive program, which had the low, fixed load of only three items to remember. However, Hyer et al. (2016) did not use the Petersen/Winblad definition of MCI, and their sample is drawn from a community population and classified with MCI according to psychometric study criteria. These differences defining MCI, and thereby divergence in the measurement of objective impairment, constitute a problem when comparing studies. This may account for the different results between the studies, rather than the differences in the effects of two types of working memory intervention.

A study of MCI participants from the Netherlands used the Cogmed software in a proof-of-principle study, but they did not compare the adaptive training program with a placebo program. Instead, they investigated the effects of adaptive training on MCI individuals as part of a larger study. They found that cognitively healthy older adults showed a tendency toward improved performance after Cogmed training on an episodic memory task (Rey Auditory Verbal Learning Test/RAVLT, similar to CVLT-II), while MCI patients showed impaired performance on this task, and several MCI patients even demonstrated declined performance at the 3 month follow up. The authors discussed that this is an expected finding, as MCI patients are known to be at risk for progression to dementia due to the underlying neurodegenerative disease, including AD (Vermeij et al., 2016). Our finding that there were no significant difference in training effect between adaptive and non-adaptive training does not necessarily represent a lack of effect, since MCI patients are expected to have degenerative neural pathology and therefore also is expected to deteriorate cognitively. As noted by Vermeij and colleagues and a large body of research, the possibility of a degenerative pathological condition in the brain affecting cognitive function is greater in patients with MCI than in a normal population (Petersen et al., 1999, 2018; Petersen, 2009; Saunders and Summers, 2010; Bondi and Smith, 2014; Vermeij et al., 2016). Therefore, our results should be interpreted with this in mind: a successful result of the adaptive cognitive intervention could also be to prevent decline and maintain status quo, rather than to expect statistically improvement in cognitive function.

### Strengths and Limitations

The major strength of this study is the randomized double-blinded design with an actively training (non-adaptive version of program) control group. The computerized cognitive tasks appeared identical on the computer screens for the participants of both training groups. Another strength is that the same experienced neuropsychologist, blinded to group allocation, conducted all the neuropsychological assessments, in order to avoid inter-rater disagreement and to minimize the risk of unsystematic errors in the test administration that could affect results in multiple test sessions. We also had a relatively high retention rate, with 78.5% in the adaptive group and 79% in the non-adaptive group completing the Cogmed training. In addition, standardized, internationally renowned neuropsychological tests were applied. Furthermore, the primary outcome measures (working memory domain) were based on tests with little or no test–retest effects. Lastly, we had a well-defined study population, consisting of patients already diagnosed with MCI in hospital-based Memory Clinics by experienced multidisciplinary teams using national assessment and diagnostic guidelines (Norcog).

A limitation of the study is the relatively small sample size, but to our knowledge, this is the largest study on MCI patients recruited from memory clinics that has implemented the Cogmed training protocol. Despite the randomization, the relatively small number of participants in the two groups might have had different levels of functioning at baseline. It is well-known and debated that the concept of MCI may capture a heterogeneous group, including some people in a prodromal state to dementia and others who may not develop dementia (Chertkow et al., 2007). The sum of heterogeneities in each individual, resulting from the interactions between three variables, brain pathology, cognitive impairment and cognitive reserve, might have been different in the groups. This difference also might have distorted the intervention effect. Lastly, participant selection might be another bias since the patients that consented to the intervention might have been highly motivated, had high grade of self-efficacy or other traits that might not be representative of all MCI patients. However, this is a common problem in clinical research.

In this study, we investigated the effects of working memory training on untrained tasks of working memory and other cognitive domains. However, the focus on neuropsychological outcomes alone may posts limitations to the study, as APS recommended that outcomes ideally also should include measures of real-world performances (Simons et al., 2016; van Heugten et al., 2016).

### Implications

Engagement in cognitive training may for 40 min a day, five times a week, in 5 weeks may have led to less time for other potential cognitively healthy activities as physical training, social gatherings and so on. Compared to pharmacological interventions, computerized cognitive training has generally been considered free from aversive effects. However, the training may have had costs in the form of frustration of not mastering the computer, feeling of failure, and disappointment if the program was not perceived as meaningful or helpful despite the effort despite the effort invested.

More research is needed to increase knowledge regarding the type of training paradigms, frequency and duration of training, that may be most effective, and what is realistic to expect as beneficial outcomes, in cognitive interventions in MCI patients.

## Conclusion

Our study investigated the effectiveness of adaptive working memory training against placebo training. No difference were found between the two types with regard to training outcome. Within groups or *post hoc* analyses were not performed due to the stringent RCT design. Within group training effects will be explored in another paper. The hypothesis that the adaptive training program would lead to greater improvements on the primary and secondary outcome measures compared to the non-adaptive training program was not supported.

## Ethics Statement

We performed a double blinded, placebo (active)-controlled, randomized clinical trial approved by the Norwegian Regional Committee for medical and health research ethics, South-Eastern region (2013/410/REK Sør-øst). Oral and written informed consents were obtained from each participant.

## Author Contributions

MF: conception and design of the study’s neuropsychological part, data collection, data analysis and interpretation, drafting the article, critical revision of the article and final approval of the version to be published. HH: conception and design of the study, data collection, critical revision of the article and final approval of the version to be published. SH: conception and design of the study, data collection, data analysis and interpretation, drafting the article, critical revision of the article and final approval of the version to be published. LC: conception and design of the study, data interpretation, critical revision of the article and final approval of the version to be published. AE: data collection and critical revision of the article and final approval of the version to be published. KB: data analysis, critical revision of the article and final approval of the version to be published. AP: conception and design of the study’s statistical part, data analysis, critical revision of the article and final approval of the version to be published. B-OM: data collection and critical revision of the article and final approval of the version to be published. A-BK: data collection and critical revision of the article and final approval of the version to be published. IU: input on the study design, data collection and critical revision of the article and final approval of the version to be published. TL: data collection and critical revision of the article and final approval of the version to be published. JS: conception and design of the study, data collection, data analysis and interpretation, critical revision of the article and final approval of the version to be published. GL: P.I of the study, conception and design of the study, data collection, data analysis and interpretation, critical revision of the article and final approval of the version to be published.

## Conflict of Interest Statement

The authors declare that the research was conducted in the absence of any commercial or financial relationships that could be construed as a potential conflict of interest.

## Footnotes

1 http://www.randomizer.org

2 http://www.matrics.ucla.edu/index.shtml

3 https://www.cogmed.com/
