# Supplementary material for: Adaptive Computerized Working Memory Training in Patients With Mild Cognitive Impairment. A Randomized Double-Blind Active Controlled Trial
Source: Front Psychol. 2019 Apr 12;10:807. doi: 10.3389/fpsyg.2019.00807 (PMC6473070; doi:10.3389/fpsyg.2019.00807)
Supplement: Supplementary file 1 [file Data_Sheet_1.pdf]

Description of the tasks included in Cogmed® Robomemo

Visual data link

A number of lamps will be highlighted in succession. The subject needs to remember in which order they came on. When the program says “Your turn,” the subject should click on the lamps in the same order as demonstrated.

Asteroids

A number of moving asteroids will light up in succession. The subject needs to remember the order in which the shapes lit up. When the program says “Your turn,” the subject should click on the shapes in the same order they lit up.

Rotating data link

A number of lamps will be highlighted in succession. The subject needs to remember the order in which they came on. Before the subject gives an answer, the entire panel will rotate 90 degrees. When the program says “Your turn,” the subject should click on the circles in the same order in which they came on, but he/she has to remember that the panel has rotated 90 degrees. The subject has to click on the circles in the right order, although they are now in new positions.

3D Cube

A number of panels will light up in different colors in succession. At the same time, the cube is turning toward each panel that light up. The subject needs to remember the order in which they lit up. When the program says “Your turn,” the subject should click on the panels in the same order.

Input module

A number of digits will be read out loud in succession. The subject needs to listen carefully and try to remember the order in which they were read. When the program says “Your turn,” the subject should click on the numbered buttons in the reverse order. Example: The digits “3, 7” will be read out loud, and the subject should click on “7, 3.”

Input module with lid

This is a different version of the input module task. The numbers are read aloud; however, the subject cannot see the numbered buttons as they are read. The numbers will appear when it is the subject's turn to click on the numbered buttons in reverse order.

Sorter

Certain boxes will be highlighted and numbers will be revealed. They will then disappear. When the program says “Your turn,” the subject should start by clicking on the box that contains the number 1, then the box that contains the number 2, 3, and so on, in numerical order.

Stabilizer

Certain letters will be read aloud. When a letter is read, it will be displayed in the middle of the circle, and at the same time, a corresponding light will light. After all the letters have been read, one of them will be displayed once again in the middle. The subject needs to remember which light came on when he/she heard that particular letter. The subject responds by clicking on the correct light.

Decoder

Certain letters will be read aloud. At the same time, the letter will light up. The subject needs to remember the letters he/she hears and then select the letters by clicking on them. Example: These letters will be heard: “D, P, E.” The first letter is “D”—you have to select that letter from the 3 options next to the first light. At the next light, you must select “P,” the second letter. Finally, you must select the “E” from the choices next to the third light.

Data room

Some of the lamps in a 3-dimensional room will lighten up. The subject needs to remember the order and then click on the lamps in the order that they lit up.

Space whack

Monsters will randomly appear in craters. Before they appear they let out a little cloud of gas, and the subject needs to remember the order of the gas clouds to be able to hit the monsters in the heads when they appear. It is important that the subject waits until all gas clouds have been shown and then be prepared to hit each monster by standing with the pointer above each crater.

Rotating

The subject will see some circles rotating. The circles will light up in a specific order. The circles will also move, so the subject needs to keep track of their initial position. The subject then clicks on the circles in the same order, although they are now in new positions.

Courtesy of www.cogmed.com .
