# Supplementary material for: Adaptive Computerized Working Memory Training in Patients With Mild Cognitive Impairment. A Randomized Double-Blind Active Controlled Trial
Source: Front Psychol. 2019 Apr 12;10:807. doi: 10.3389/fpsyg.2019.00807 (PMC6473070; doi:10.3389/fpsyg.2019.00807)
Supplement: Supplementary file 2 [file Table_1.pdf]

Supplemental:

Table 1S. Means and Standard Deviations of the neuropsychological test results on the subtests for the placebo training group and adaptive training group				
Placebo (n=34)<br>Adaptive (n=34)	Mean Scores Over Time<br>Sig.(2-tailed)			
**Test scores**	**1-month Post Training (SD)**	**Sig.**	**3-month Follow up (SD)**	**Sig.**
**Digit span backwards, longest number of digits remembered**				
placebo	4.00 (0.98)	0.39	3.79 (1.20)	0.14
pdaptive	4.24 (1.25)		4.24 (1.23)	
**Letter-number sequencing**				
placebo	7.74 (2.71)	0.32	7.26 (2.32)	0.15
adaptive	8.38 (2.69)		8.18 (2.88)	
**Spatial span backwards**				
placebo	6.44 (1.77)	0.44	5.82 (1.89)	0.30
adaptive	6.82 (2.24)		6.29 (1.81)	
**Digit span forward, longest number of digits remembered**				
placebo	5.41 (1.04)	0.73	5.32 (1.09)	0.91
adaptive	5.50 (1.05)		5.35 (1.07)	
**Spatial span forward**				
placebo	6.35 (1.85)	0.31	6.38 (1.68)	0.25
adaptive	6.85 (2.21)		7.38 (1.90)	
**CVLT-II Trial 1**				
placebo	4.00 (1.63)	0.57	4.68 (2.13)	0.62
adaptive	4.52 (1.75)		4.75 (1.62)	
**CVLT-II Trial B**				
placebo	3.70 (1.40)	0.78	3.97 (1.47)	0.86
adaptive	3.76 (1.56)		3.97 (1.56)	
**Color Word Inference Test Color Naming (seconds)**				
placebo	40.50 (18.27)	0.14	41.47 (17.92)	0.74
adaptive	40.91 (12.35)		41.73 (12.48)	
**Color Word Inference Test Word reading (seconds)**				
placebo	31.18 (17.04)	0.23	32.18 (17.54)	0.10
adaptive	28.73 (12.89)		29.36 (12.52)	
**Rey complex figure, short delay recall**				
placebo	12.84 (7.64)	0.79	14.15 (9.69)	0.12
adaptive	14.88 (7.90)		17.12 (8.45)	
**WMS-III Faces, short delay recall**				
placebo	34.47 (5.84)	0.54	34.44 (8.82)	0.36

adaptive	34.44 (7.98)		35.88 (6.64)	
**Rey complex figure, long delay recall**				
placebo	13.19 (8.01)	0.81	14.29 (9.55)	0.36
adaptive	15.53 (8.54)		16.00 (8.91)	
**WMS-III Faces, long delay recall**				
placebo	33.35 (7.33)	0.23	32.82 (8.36)	0.69
Adaptive	34.18 (6.10)		35.05 (7.10)	
**WMS-III Logical memory, short delay recall**				
placebo	28.85 (10.94)	0.36	29.65 (13.72)	0.52
adaptive	29.74 (12.88)		32.12 (14.03)	
**CVLT-II Trial 5**				
placebo	7.85 (3.03)	0.36	8.26 (2.84)	0.16
adaptive	9.24 (3.50)		9.09 (3.49)	
**CVLT-II Total learning**				
placebo	32.00 (10.18)	0.27	34.13 (12.37)	0.14
adaptive	37.57 (12.83)		36.84 (14.08)	
**CVLT-II Short delay free recall**				
placebo	4.97 (3.61)	0.43	5.10 (3.86)	0.90
adaptive	7.00 (3.92)		6.91 (3.88)	
**CVLT-II Short delay cued recall**				
placebo	6.41 (3.39)	0.23	7.03 (3.56)	0.56
adaptive	8.48 (3.69)		8.19 (3.31)	
**Logical memory, long delay recall**				
placebo	14.15 (9.48)	0.66	14.50 (10.98)	0.99
adaptive	17.33 (8.77)		19.29 (10.86)	
**CVLT-II Long delay free recall**				
placebo	4.61 (4.06)	0.54	5.52 (4.38)	0.65
adaptive	6.91 (4.52)		6.34 (4.72)	
**CVLT-II Long delay cued recall**				
placebo	4.81 (4.02)	0.29	5.81 (4.36)	0.79
adaptive	7.56 (4.60)		7.14 (4.00)	
**CVLT-II Total hits recognition trial**				
placebo	11.73 (5.38)	0.78	13.03 (4.10)	0.23
adaptive	13.06 (5.05)		12.69 (4.21)	
**Rey figure Copy Trial**				
placebo	33.88 (2.61)	0.06	33.24 (4.70)	0.51
adaptive	32.65 (4.26)		32.36 (4.37)	
**Verbal Fluency Test Letter fluency**				
placebo	40.47 (17.79)	0.09	39.06 (16.59)	0.46
adaptive	43.58 (11.91)		42.61 (11.39)	
**Verbal fluency test Category fluency**				

placebo	33.12 (10.56)	0.71	32.50 (10.55)	0.81
adaptive	36.18 (10.45)		36.21 (12.04)	
**Verbal Fluency test Category Switching**				
placebo	10.38 (4.28)	0.38	10.74 (4.64)	0.41
adaptive	11.24 (4.92)		11.13 (4.30)	
**Color Word Inference Test Inhibition**				
placebo	89.50 (43.60)	0.02	82.85 (40.47)	0.72
adaptive	79.67 (33.12)		80.09 (31.38)	
**Color Word Inference Test Inhibition Switching**				
placebo	106.62 (43.98)	0.19	107.74 (43.41)	0.82
adaptive	96.55 (39.41)		93.48 (38.22)	

Table 2S.
Mean difference between group. P-values are from Linear mixed model for repeated measurements

**Test scores**	**One month after training**				**Four months after training**		
	Group difference	p-value	95% CI		Group difference	p-value	95% CI
**Working memory domain (main outcome):**							
Digit span backwards, longest number of items remembered	0.176	0.431	(-0.262-0.615)		0.396	0.077	(-0.042-0.835)
Letter-number sequencing	-0.138	0.735	(-0.937-0.661)		0.174	0.668	(-0.625-0.975)
Spatial span backwards	0.168	0.673	(-0.615-0.953)		0.243	0.543	(-0.541-1.028)
**Attention domain:**							
Digit span forward, longest number of items remembered	0.041	0.84	(-0.374-0.456)		-0.025	0.906	(-0.440-0.456)
Spatial span forward	0.054	0.888	(-0.699-0.808)		0.436	0.262	(-0.325-1.198)
CVLT-II Trial 1	0.473	0.199	(-0.248-1.195)		0.109	0.771	(-0.628-0.847)
CVLT-II Trial B	0.793	0.147	(-0.277-1.864)		0.265	0.632	(-0.821-1.353)
**Processing speed domain:**							
Color Word Inference Test Color Naming (seconds)	-0.279	0.838	(-2.950-2.392)		-0.303	0.824	(-2.974-2.368)
Color Word Inference Test Word reading (seconds)	0.884	0.546	(-1.985-3.754)		-0.016	0.991	(-2.882-2.849)
**Visual learning, short delay recall**							

Rey complex figure, short delay recall	-0.201	0.875	(-2.727-2.324)		-0.208	0.873	(-2.761-2.345)
WMS-III Faces, short delay recall	-0.871	0.528	(-3.578-1.834)		0.641	0.642	(-2.064-3.347)
**Visual memory, long delay recall**							
Rey complex figure, long delay recall	0.423	0.726	(-1.941-2.788)		-0.894	0.462	(-3.279-1.490)
WMS-III Faces, long delay recall	0.277	0.853	(-2.663-3.219)		1.750	0.240	(-1.169-4.640)
**Verbal learning, short delay recall domain**							
WMS-III Logical memory, short delay recall	-1.399	0.452	(-5.046-2.248)		-0.416	0.823	(-4.066-3.234)
CVLT-II Trial 5							
CVLT-II Total learning	3.230	0.100	(-0.616-7.076)		0.903	0.648	(-2.972-4.780)
CVLT-II Short delay free recall	1.368	**0.003**	(0.468-2.269)		1.286	**0.006**	(0.373-2.200)
CVLT-II Short delay cued recall	1.091	0.057	(-0.030-2.213)		0.365	0.527	(-0.767-1.498)
**Verbal memory, long delay recall domain**							
Logical memory, long delay recall	-0.983	0.510	(-3.907-1.940)		0.106	0.943	(-2.803-3.016)
CVLT-II Long delay free recall	1.962	**0.005**	(0.600-3.325)		0.565	0.420	(0.808-1.940)
CVLT-II Long delay cued recall	1.383	0.041	(0.057-2.710)		0.188	0.785	(-1.163-1.539)
**Verbal memory, recognition domain**							
CVLT-II Total hits recognition trial	0.733	0.399	(-0.973-2.441)		-0.943	0.306	(-2.747-0.861)
CVLT-II Total false positive	-0.781	0.473	(-2.918-1.355)		-0.682	0.546	(-2.900-1.534)
**Executive function domain**							
Rey figure Copy Trial	-0.795	0.303	(-2.308-0.716)		-0.632	0.413	(-2.148-0.883)
Verbal Fluency Test Letter fluency	2.008	0.280	(-1.633-5.651)		1.953	0.294	(-1.694-5.600)
Verbal fluency test Category fluency	0.674	0.668	(-2.410-3.760)		0.979	0.535	(-2.111-4.069)
Verbal Fluency test Category Switching	-0.079	0.920	(-1.617-1.459)		-0.918	0.247	(-2.472-0.635)
Color Word Inference Test, Inhibition	-4.629	0.212	(-11.905-2.646)		-4.282	2.250	(-3.018-11.584)
Color Word Inference Test, Inhibition Switching	-6.094	0.296	(-17.517-5.333)		-8.439	0.148	(-19.874-2.995)
